# Oncomodulin: The Enigmatic Parvalbumin Protein

**DOI:** 10.3389/fnmol.2019.00235

**Published:** 2019-10-09

**Authors:** Leslie K. Climer, Andrew M. Cox, Timothy J. Reynolds, Dwayne D. Simmons

**Affiliations:** ^1^Department of Biology, Baylor University, Waco, TX, United States; ^2^Department of Psychology and Neuroscience, Baylor University, Waco, TX, United States; ^3^Biomedical Sciences Program, Baylor University, Waco, TX, United States

**Keywords:** EF-hand Ca-binding protein ++, cochlea, hair cell, macrophage, beta parvalbumin, phylogeneticanalysis

## Abstract

EF-hand Ca^2+^-binding protein family members, α- and β-parvalbumins have been studied for decades. Yet, considerable information is lacking distinguishing functional differences between mammalian α-parvalbumin (PVALB) and oncomodulin (OCM), a branded β-parvalbumin. Herein, we provide an overview detailing the current body of work centered around OCM as an EF-Hand Ca^2+^-binding protein and describe potential mechanisms of OCM function within the inner ear and immune cells. Additionally, we posit that OCM is evolutionarily distinct from PVALB and most other β-parvalbumins. This review summarizes recent studies pertaining to the function of OCM and emphasizes OCM as a parvalbumin possessing a unique cell and tissue distribution, Ca^2+^ buffering capacity and phylogenetic origin.

## Introduction

**Figure 1:**
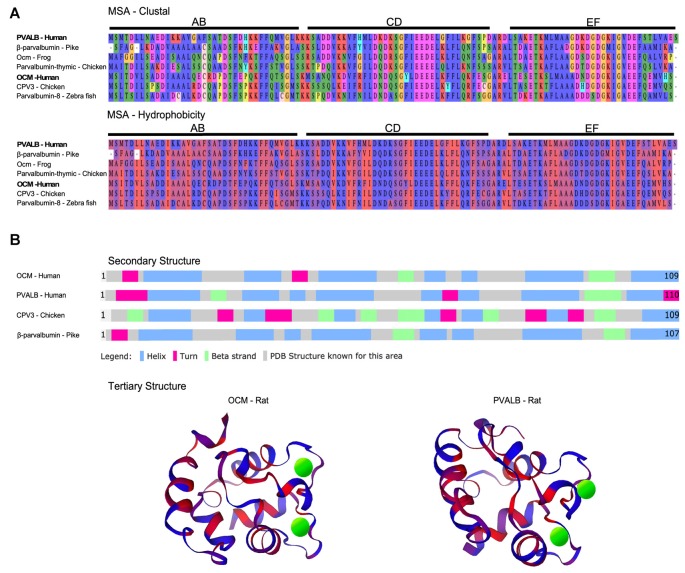
Multiple sequence alignment (MSA) and structural comparison of oncomodulin (OCM) to other parvalbumins.** (A)** Seven representative parvalbumin protein sequences were aligned using the MUSCLE algorithm *via* MPI Bioinformatics Toolkit (Edgar, 2004; Zimmermann et al., 2018). The sequences are grouped together by MUSCLE based on their pairwise sequence similarities to generate the final MSA. Thus, the order of the proteins in the MSA reflects how similar the sequences are in terms of structure, evolutionary relationships, and/or function. The final MSA is shown in two color schemes which denote amino acids based on biophysical (clustal) and hydrophobic properties. Biophysical characteristics are organized using a Clustal2 coloring scheme. Yellow = proline; orange = glycine; red = negatively charged; fuchsia = positively charged; green and blue = polar; peach = cysteine. Hydrophobicity color scheme shows hydrophilic residues in blue and hydrophobic residues in red. **(B)** Secondary structures for four representative parvalbumins were obtained from Uniprot.org. Tertiary structures are colored based on predicted hydrophobic properties and were obtained from the Protein Database—Europe *via* Uniprot.org. Green spheres in tertiary structures represent Ca^2+^ ions.

Oncomodulin (OCM) is a small EF-hand Ca^2+^-binding protein (CaBP) of approximately 12 kDa belonging to the parvalbumin family. As shown in Figure 1, mammalian OCM is the β isoform of parvalbumin and shares at least 53% sequence identity with α-parvalbumin (PVALB; Berchtold, 1989). It has an unusually restrictive post-embryonic expression pattern in mammals: limited mostly to subsets of sensory hair cells in the inner ear and more recently found in certain subtypes of immune cells. In addition to OCM, the mammalian inner ear has other major EF-hand CaBPs including PVALB, calbindins (CB-D28k), calretinin (CB-D29k), calmodulin, and the S100 proteins (see Table 1 for respective gene identifiers). There also may be other EF-hand CaBPs, such as the recent discovery of the penta-EF-hand CaBP Sorcin (*Sri*), in the inner ear (Ranum et al., 2019). Although there have been excellent reviews focused more generally on the family of EF-hand CaBPs and its members (Pauls et al., 1996; Schwaller et al., 2002; Schwaller, 2009, 2010, 2014; Permyakov et al., 2017), this review is the first dedicated to OCM in light of significant new findings.

**Table 1:** Protein expression of select Ca^2+^-binding proteins.

Protein	Gene ID	Tissue Expression	Source
**Mammalian parvalbumins**			
Parvalbumin (PVALB)*	PVALB*	Fast-twitch skeletal muscle	Baron et al. (1975), Heizmann et al. (1982), Föhr et al. (1993), Hou et al. (1993), Westerblad and Allen (1994) and Uhlén et al. (2015)
		Mature inner hair cells	Bergeron et al. (2005) and Hackney et al. (2005)
		Inhibitory and glutamatergic neurons in the brain	Baron et al. (1975), Föhr et al. (1993), Caillard et al. (2000), Schwaller et al. (2002), Csillik et al. (2010), Shang et al. (2015) and Uhlén et al. (2015)
		Kidney	Baron et al. (1975), Föhr et al. (1993) and Uhlén et al. (2015)
		Thymus and Lung	Föhr et al. (1993) and Uhlén et al. (2015)
		Heart	Föhr et al. (1993)
Oncomodulin (OCM)*	OCM*	Mature outer hair cells	Sakaguchi et al. (1998), Hackney et al. (2005) and Simmons et al. (2010)
		Vestibular hair cells	Simmons et al. (2010) and Hoffman et al. (2018)
		Infiltrative-macrophages	Yin et al. (2009) and Kurimoto et al. (2010)
		Macrophages (post-injury)–Pancreas	Siawaya et al. (2013)
		Placenta	MacManus et al. (1985)
**Sampling of non-mammalian parvalbumin**			
Parvalbumin (tPvalb)^∨^	none^∨^	Skeletal muscle, Gill, Heart, Brain, Kidney, Ovary	Lee et al. (2007)
Parvalbumin (PV)^†^	none^†^	Brain and Muscle	Schwartz and Kay (1988)
Thymic chicken parvalbumin 3 (CPV3)^∧^	OCM^∧^	Thymic cortex	Hapak et al. (1994, 1996)
Avian thymic hormone (ATH)^∧^	none^∧^	Thymic cortex	Brewer et al. (1989, 1990), Barger et al. (1991) and Hapak et al. (1996)
**Sampling of other mammalian EF-hand CaBP**			
Calbindin (CB-D28k)*	Calb1*	Retinal Neurons	Mojumder et al. (2008)
		Kidney and Intestines	Wood et al. (1988) and Armbrecht et al. (1989)
		Brain	McIntosh et al. (1986) and Wood et al. (1988)
		Bone	Faucheux et al. (1998) and Bellido et al. (2000)
		Pancreas	Berdal et al. (1996)
		Teeth	Sooy et al. (1999) and Onishi et al. (2008)
		Placenta	Koo et al. (2012)
Calretinin (CB-D29k)*	Calb2*	Brain	Jungenitz et al. (2014)
		Ovary	Bertschy et al. (1998)
		Retinal Neurons	Jeon and Jeon (1998)

*Indicates Protein and Gene ID are from Homo sapians (Human), ^∨^indicates Protein and Gene ID are from Oreochromis mossambicus (Tilapia), ^†^indicates Protein and Gene ID are from Xenopus laevis (African clawed frog), ^∧^inidicates Protein and Gene ID are from Gallus gallus (Chicken).

The term “oncomodulin” is derived from the initial discovery of OCM in cancerous tissue as an oncoprotein and from its similarity to calmodulin as a CaBP (Stavrou et al., 1971; MacManus et al., 1982, 1985). Initially, OCM was considered oncogenic due to lack of evidence of any expression in normal post-embryonic tissue. However, decades after its initial discovery, OCM was identified as a major protein in sensory cells of the guinea pig cochlea (Senarita et al., 1995; Thalmann et al., 1995; Henzl et al., 1997). Guinea pig OCM shares 90%, 92%, and 98% identity with mouse, rat, and human OCM, respectively (Henzl et al., 1997). Later studies of the rat inner ear suggested that OCM was expressed mostly in cochlear outer hair cells (OHCs) and possibly within vestibular hair cells (Sakaguchi et al., 1998). More recently, OCM was validated in OHCs across mammalian species, in vestibular hair cells (Simmons et al., 2010; Tong et al., 2016; Hoffman et al., 2018), and in immune cells (Yin et al., 2006, 2009; Kurimoto et al., 2010; Siawaya et al., 2013). Indeed, the unique cell and tissue distribution (Tables s 1–3) displayed by OCM begs the question: what specialized role or function does OCM have that other mobile Ca^2+^ buffers do not?

**Table 2:** Protein expression of select Ca^2+^-binding proteins in rat cochlear inner (IHCs) and outer hair cells (OHCs).

Protein	Cell type	P0-P5	P6-P10	P11-P20	P21-adult	Reference
OCM	OHC	0–0.5 ng/μg	2.0 ng/μg	2.4 ng/μg	1.8 ng/μg	Yang et al. (2004)
		***	~0–250 μM	~1–1.6 mM	2–3 mM	Hackney et al. (2005)
	IHC	none	none	***	none	Yang et al. (2004)
		***	<50 μM	0 μM	0 μM	Hackney et al. (2005)
∣rule
PVALB	OHC	present	diminishing	***	none	Yang et al. (2004)
		***	<35 μM	138 μM	100–300 μM	Hackney et al. (2005)
	IHC	present	increasing	***	abundant	Yang et al. (2004)
		***	~80–110 μM	89 μM	~150 μM	Hackney et al. (2005)
∣rule
CB-D28k	OHC	***	***	***	***	Yang et al. (2004)
		***	~400 μM	196 μM	15–230 μM	Hackney et al. (2005)
	IHC	***	***	***	***	Yang et al. (2004)
		***	~400 μM	57 μM	0 μM	Hackney et al. (2005)
∣rule
CB-D29k	OHC	***	***	***	***	Yang et al. (2004)
		***	<35 μM	35 μM	~30–60 μM	Hackney et al. (2005)
	IHC	***	***	***	***	Yang et al. (2004)
		***	~19 μM	19 μM	~50 μM	Hackney et al. (2005)

Yang et al. (2004) is listed as ng of OCM protein/μg of dry weight organ of Corti. Hackney et al. (2005) is listed as Molar concentration of protein per cell. ***Timepoint not analyzed.

**Table 3:** Protein Expression of select Ca^2+^-binding proteins in mouse and rat vestibular hair cells.

Protein	E17–18	P0	P3–4	P10	2–6 weeks	6–8 months	>10 months*	Reference
**Utricle**
OCM	present	***	present	present	present	diminishing	absent	Simmons et al. (2010)
PVALB	absent	present	present	***	***	***	***	Zheng and Gao (1997)
CB-D28k	present	absent	absent	absent	***	***	***	Buckiová and Syka (2009)
CB-D29k	present	present	present	present	absent	***	***	Dechesne et al. (1994)
**Saccule**
OCM	present	***	present	***	***	***	absent	Simmons et al. (2010)
PVALB	absent	present	present	***	***	***	***	Zheng and Gao (1997)
CB-D28k	present	absent	absent	absent	***	***	***	Buckiová and Syka (2009)
CB-D29k	***	***	***	***	***	***	***	Dechesne et al. (1994)
**Cristae**
OCM	present	present	present	***	present	diminishing	absent	Simmons et al. (2010)
PVALB	absent	present	present	***	***	***	***	Zheng and Gao (1997)
CB-D28k	present	absent	absent	absent	***	***	***	Buckiová and Syka (2009)
CB-D29k	present	present	present	present	absent	***	***	Dechesne et al. (1994)

*Data in Table 3 represents immunoreactivity of OCM except at 10-months where mRNA expression is described. ***Timepoint not analyzed.

**Figure 2:**
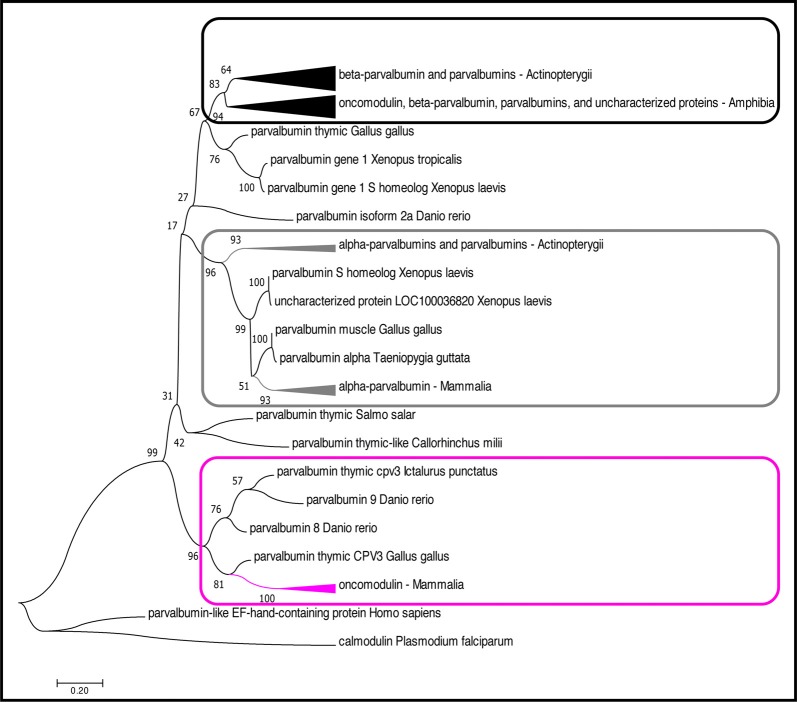
Phylogenetic analysis of OCM and PVALB homologs. The evolutionary history was inferred by using the Maximum likelihood (ML) method based on the (Le and Gascuel, 2008) model. The boxes (magenta, black, gray) denote three distinct lineages of parvalbumin proteins. The magenta box indicates the mammalian OCM branch. The gray box indicates the α-parvalbumin branch. The black box indicates the β-parvalbumins from lower vertebrates and OCM from *Xenopus subsp*. The tree with the highest log likelihood (−4,296.92) is shown. The percentage of trees in which the associated taxa clustered together is shown next to the branches. The tree is drawn to scale, with branch lengths measured in the number of substitutions per site. The analysis involved 51 amino acid sequences. All positions with less than 95% site coverage were eliminated. That is, fewer than 5% alignment gaps, missing data, and ambiguous bases were allowed at any position. There was a total of 109 positions in the final dataset. Evolutionary analyses were conducted in MEGA7 (Kumar et al., 2016). The arrowheads on the branches represent a cluster of sequences that have been compiled and represented by a single node for viewing simplicity. The arrowhead size is proportional to the number of sequences within the cluster.

There are only a limited number of studies on the function of OCM largely due to its very restricted temporal and spatial expression patterns. Also, only a few OCM studies have addressed: intracellular concentration, affinity for metal ions, mobility, and Ca^2+^-sensing capacity. Whether a single function for OCM exists, similar to its α isoform, PVALB, is not known. In addition to its limited expression in mammals, the variety and number of β-parvalbumin isoforms across vertebrate species make it very difficult to draw conclusions about OCM function (Table 1, Figures 1, 2). The mosaic of work on OCM spans more than five decades of research and draws from the fields of biochemistry, molecular biology, bioinformatics and neurobiology. This review seeks to clarify phylogenetic relationships of OCM with other β-parvalbumins, to review what we know of its Ca^2+^-buffering capacities in comparison to other EF-hand CaBPs, and to propose two different models of OCM function in mammals in the hopes of providing a more holistic view of OCM.

## Phylogenetic Analysis of Oncomodulin

Although OCM shares many of the essential features of β-parvalbumins (described below), we show that mammalian OCM does not share a common phylogenetic lineage with lower vertebrate β-parvalbumins of the parvalbumin family (Figure 2). While there have been multiple phylogenetic analyses performed using parvalbumin sequences, none of them specifically examined the relationship of OCM within other parvalbumins (Pauls et al., 1996). The reason for this is two-fold. First, recent phylogenetic analyses of parvalbumins have only been conducted using fish parvalbumins (Lee et al., 2007; Perez-Gordo et al., 2011). Second, phylogenetic analyses that include parvalbumin sequences outside of fish were performed mostly in the 1970s and 1980s, and thus used a limited number of parvalbumin sequences (Pechere et al., 1973; Goodman and Pechére, 1977; Cavé et al., 1982; Maeda et al., 1984; Moncrief et al., 1990). For example, the most recent phylogenetic analysis that included mammalian OCM used only 23 parvalbumin sequences, two of which were from mammals, and none from birds (Moncrief et al., 1990). Since then, numerous advances have improved the field of bioinformatics to allow quick and more detailed construction of sequence alignments, tests of models for constructing a phylogenetic tree, and validation of the tree. Additionally, a greater number of complete parvalbumin sequences has been discovered. Here, a bootstrapped-maximum likelihood (ML) phylogenetic tree was created using 55 non-redundant homologous parvalbumin sequences representing fish (*n* = 23), birds (*n* = 4), amphibians (*n* = 17), and mammals (*n* = 11) in order to better elucidate how OCM fits within parvalbumins (Appendix).

Based on the multiple sequence alignment (MSA) in Figure 1 and phylogenetic analysis in Figure 2; it is apparent that mammalian OCM is not phylogenetically similar to β-parvalbumins from lower vertebrates. The mammalian OCM branch of the tree (magenta box) is grouped separately from all the β-parvalbumins (black box) used in this analysis (*n* = 7). Contrarily, the α-parvalbumin branch in Figure 2 (gray box) clearly shows that mammalian α-parvalbumin shares a conserved phylogeny with α-parvalbumins from lower vertebrates. Previously, there was no evidence to distinguish mammalian OCM from other β-parvalbumins. However, the present computational analysis shows otherwise. If the essential features of β-parvalbumins such as isoelectric point, location of cysteine residues and amino acid length were sufficient to classify mammalian OCM with other β-parvalbumins, then they would share much of the same sequence phylogeny just as mammalian α-parvalbumin does with α-parvalbumins from lower vertebrates. Additionally, Figure 2 demonstrates that the relationship of mammalian OCM and an OCM named in the frog are quite different. In Figure 2, OCM from *Xenopus subsp*. shared a higher degree of phylogeny and grouped with the β-parvalbumins from lower vertebrates rather than with mammalian OCM. On this basis, it seems that OCM in *Amphibia* was mislabeled and needs to be reclassified as another type of β-parvalbumin instead of being termed OCM. Indeed, Figure 1 shows that frog OCM has the greatest pairwise sequence similarity to β-parvalbumin from pike rather than to OCM from human. Taken all together, these analyses show for the first time that mammalian OCM is phylogenetically distinct from many, if not all, β-parvalbumins in lower vertebrates. So, while mammalian OCM by definition possesses defining traits of β-parvalbumins, this analysis shows that there are other features of the protein sequence that suggests they are not very closely related. Thus, mammalian OCM may be evolutionarily divergent from most other β-parvalbumins, especially, in lower vertebrates. If true, a new subcategory of parvalbumins would be justified. Consistent with the lack of shared phylogeny, β-parvalbumin and mammalian OCM do not exhibit similar expression patterns. While β-parvalbumins from fish and frog are expressed in a wide variety of tissue, including muscle, kidney, and brain (Gosselin-Rey et al., 1978; Sakaizumi, 1985; Brownridge et al., 2009), mammalian OCM expression is distinct and restricted to specific inner ear hair cells and some immune cells (Table 1). Furthermore, Figure 2 suggests that OCM may have evolved from specific thymic parvalbumins in lower vertebrates, namely parvalbumin thymic CPV3 from *Ictalurus punctatus* and parvalbumin thymic-like protein from *Callorhinchus milii*, based on their shared phylogeny. It is worth mentioning that there are different thymic parvalbumins which are not extremely closely related despite the shared name, e.g., CPV3 and thymic parvalbumin in chicken (Figure 2). While a more thorough examination is required to support such a hypothesis, the specialized and restricted expression pattern of mammalian OCM (Table 1) could be consistent with expression patterns of thymic parvalbumin in lower vertebrates. Further studies examining thymic parvalbumins expression in lower vertebrates are needed.

## Oncomodulin Biochemistry

EF-hand CaBPs are broadly described as mobile, or cytosolic, divalent cation buffers or sensors based on protein structural changes when bound to Ca^2+^ and selective interactions with effector proteins (Pauls et al., 1996; Kawasaki et al., 1998; Yap et al., 1999; Gifford et al., 2007; Kawasaki and Kretsinger, 2017). The Ca^2+^-binding site is comprised of a helix-loop-helix structure (Figure 1B) that is common to all EF-hand family members (Kretsinger et al., 1972; Permyakov et al., 2017). PVALB, calbindin (CB-D28k) and calretinin (CB-D29k) are EF-hand proteins are believed to act primarily as mobile Ca^2+^ buffers, while calmodulin and the S100 family of proteins act primarily as Ca^2+^ sensors (Schwaller et al., 2002; Schwaller, 2009, 2010, 2014). In general, CaBPs act intracellularly, either to sculpt the spatiotemporal aspects of cytosolic Ca^2+^ transients, or to interact with specific targets in a Ca^2+^-regulated manner. Of course, a Ca^2+^ sensor at sufficiently high enough concentration will exhibit some buffering capacity. Although a significant amount of information is known about the chemistry of non-mammalian parvalbumin isoforms, relatively little is known about the specific Ca^2+^-binding capacity of OCM (Pauls et al., 1996). Mammalian OCM shares essential features of most other vertebrate β-parvalbumins, such as, an isolectric point <5.0, a cysteine at position 19, and a length of 109 amino acids. However, based on the phylogenetic analysis presented above (Figure 2), significant caution is warranted when applying results of non-mammalian β-parvalbumin studies to mammalian OCM. First, we address what is known about the buffering capacity of OCM, and then we address its sensor capacity.

The ability of a CaBP to buffer a Ca^2+^ signal depends on the intracellular concentration of the protein, its metal ion binding affinities, and its intracellular mobility. The most direct estimate of OCM intracellular concentration comes from a study in rat using quantitative immunogold methods (Hackney et al., 2005). In this study, the authors measured the concentrations of four major EF-hand CaBPs in rat cochlear inner and outer hair cells (IHCs and OHCs), which is discussed in more detail below. Altogether, they estimated that there were 5.4 mM Ca^2+^-binding sites in apical OHCs and 6.0 mM Ca^2+^-binding sites for basal OHCs, of which OCM is the overwhelming contributor. Additionally, significant variations were observed in the distribution of CaBPs within OHCs. In particular for OCM, there was over a 3.5-fold reduction in concentration in stereocilia (969 μM) relative to apical cuticular plate (3.4 mM). In a separate study, OCM concentration was observed as high as 2.4 mg/g dry weight cochlea (Yang et al., 2004). In vertebrates, this large millimolar concentration of OCM in OHCs is comparable only to the millimolar concentration of PVALB in fast-twitch muscle fibers, where the binding of Ca^2+^ by PVALB facilitates muscle relaxation (Muntener et al., 1995; Pauls et al., 1996). Estimates of PVALB concentration in fast-twitch muscle range from 0.6 to l.0 mg/g wet muscle in rabbits (Klug et al., 1983), and 3–5 mg/g wet muscle in mouse and rat (Heizmann, 1984). The high intracellular concentration of OCM in the inner ear suggests that it is as efficient of a Ca^2+^ buffer as PVALB.

The metal ion binding capacity of OCM differs from other parvalbumins as shown in large part by the work of Henzl and colleagues. Similar to its α isoform, OCM has six α-helixes (A–F) organized into AB, CD and EF domains. The CD and EF domains are adjacent domains and contain the two Ca^2+^-binding sites (Figure 1B). OCM differs from its α isoform in its isoelectric point, C-terminal helix length, and affinity for divalent ions (Goodman and Pechére, 1977; Hapak et al., 1989; Cox et al., 1990; Moncrief et al., 1990). Henzl and Tanner (2007) determined the solution structure and peptide backbone dynamics of Ca^2+^-free and Ca^2+^-bound rat OCM. Removal of Ca^2+^ leads to structural alterations not found within PVALB. Addition of Ca^2+^ causes the C, D, and E helices to undergo substantial reorganization. Additionally, the OCM CD and EF metal ion binding sites do not show equivalent binding affinities as they do for PVALB. The EF-binding site of OCM has Ca^2+^- and Mg^2+^-binding constants of 2.5 × 10e-7 and 9.0 × 10e-3, which is similar to rat PVALB Ca^2+^- and Mg^2+^-binding constants of 1.2 × 10e-8 and 1.8 × 10e-4. However, the CD binding site of OCM has Ca^2+^- and Mg^2+^-binding constants of 1.5 × 10e-6 and 1.6 × 10e-2 (Hapak et al., 1989; Cox et al., 1990; Henzl et al., 2004). Thus, OCM differs from PVALB in that OCM has a cation binding site that will accommodate Ca^2+^ and Mg^2+^ and one that is more Ca^2+^ specific. Taken together, these studies suggest that OCM is not strictly a Ca^2+^ buffer and may be capable of acting as a Ca^2+^ sensor under the right physiological conditions, unlike PVALB.

The structural re-organization of Ca^2+^-bound OCM allows for potential effector protein interactions. Unlike calmodulin, which is known to interact with several proteins in a Ca^2+^-specific manner (Sharma and Parameswaran, 2018), OCM has no known interacting partners as there is a complete lack of experimental data. Regardless, studies to date only support OCM functioning as a cation buffer, particularly Ca^2+^ and Mg^2+^, and as a secreted, pro-regenerative protein in macrophages and neutrophils (Yin et al., 2006; Henzl and Tanner, 2007; Kurimoto et al., 2013; Tong et al., 2016). Further investigation into the sensor capacity of OCM and identification of its potential effector proteins is warranted.

## Oncomodulin in Hair Cells of the Inner Ear

### Cochlear Expression of OCM

**Figure 3:**
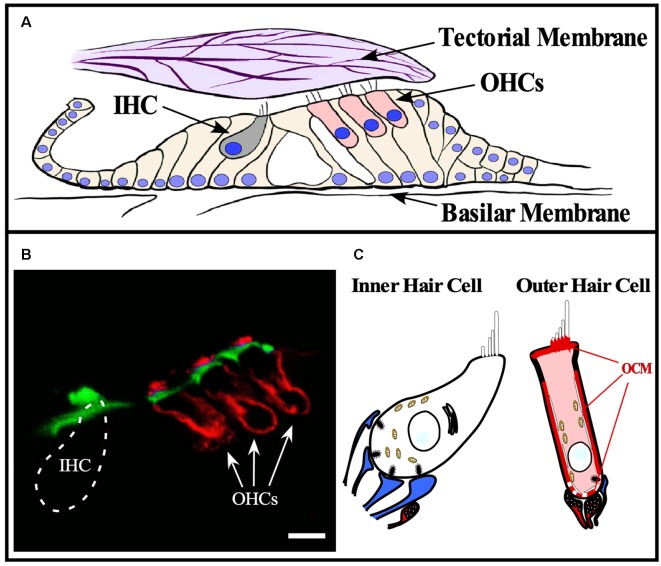
OCM expression in cochlear hair cells. OCM is expression is enriched around the basolateral membranes of outer hair cells. **(A)** Schematic of organ of Corti within the basilar membrane of the cochlea. The cochlea is a bony labyrinth that converts sound into neural impulses. This is achieved *via* two types of hair cells within the organ of Corti, IHCs and OHCs. IHCs make up a single row, and OHCs make up three consecutive rows. OHCs amplify the vibrations of the basilar membrane by interacting directly with the tectorial membrane through specialized cilia, stereocilia, that are atop both types of hair cells. IHC stereocilia do not directly interact with the tectorial membrane but respond to the vibrations amplified by OHCs and transmit the fluid distortions to the auditory nerve fibers responsible for transmitting sound. **(B)** A confocal image of OCM labeling (red) and phalloidin staining (green) in a cross-section of the mouse organ of Corti from the basal turn of the cochlea. OCM labeling preferentially localized to the basolateral membrane of OHCs and is not present in IHCs. Scale bar represents 10 μm. Modified from Simmons et al. (2010), permission granted by John Wiley & Sons, Inc., Hoboken, NJ, USA. **(C)** Schematic representation of OCM immunoreactivity in a cochlear OHC. Afferent terminals are shown in blue, efferent terminals are shown in red.

The first report linking OCM to inner ear tissues was in guinea pig by Thalmann and colleagues (Senarita et al., 1995). Subsequently, it was determined that OCM protein is expressed in only a subset of sensory cells (Thalmann et al., 1997; Sakaguchi et al., 1998). In the adult mammalian cochlea, OCM immunoreactivity is mostly restricted to the outer hair cells (OHCs; Table 2). Outer hair cells are one of two sensory hair cells in the organ of Corti, the sensory epithelium in the cochlea (Figure 3). Inner hair cells (IHCs) are primarily responsible for neurotransmission of sound stimuli directly to cochlear nerve fibers whereas the OHCs amplify the sound-induced vibrations *via* a Prestin (Slc26a5)-based electromotility mechanism. Yang et al. (2004) found both OCM mRNA expression and immunoreactivity was limited to OHCs in the rat cochlea. Using high-resolution immunogold labeling techniques in rats, Hackney et al. (2005) observed OCM at near background levels in IHCs and much higher levels in OHCs. The density of gold particles was calibrated by comparison with immunogold labeling of a section of aldehyde-fixed gel containing a known amount of the protein in order to describe results in molar concentrations. In P26 rats, the CaBP concentrations were near 2–3 mM for OCM, 230 μM and 15 μM for CB-D28k, roughly 40 μM and 65 μM for CB-D29k, and near 300 μM and 100 μM for PVALB, in apical and basal OHCs respectively (Hackney et al., 2005). They also reported OCM localized to the OHC cuticular plate and hair cell cytoplasm but not mitochondria. Using high resolution and high gain confocal microscopy in both mice and rat cochlear tissues, Simmons et al. (2010) suggested that OCM preferentially localizes to the lateral membrane, the basal portion of the hair bundle and basal pole opposite efferent terminals (Figures 3B,C). They also reported OCM localized to the cuticular plate at the base of the stereociliary hair bundle. Such localization studies led Simmons et al. (2010) to hypothesize that OCM could work as a specialized Ca^2+^ buffering system suited to the unique action of OHCs in cochlear amplification and transduction. Irrespective of the methods used, IHCs have either no or little OCM expression and the vast majority of OCM expression in the cochlea is found in the OHCs (Table 2, Figure 3C).

### Vestibular Expression of OCM

**Figure 4:**
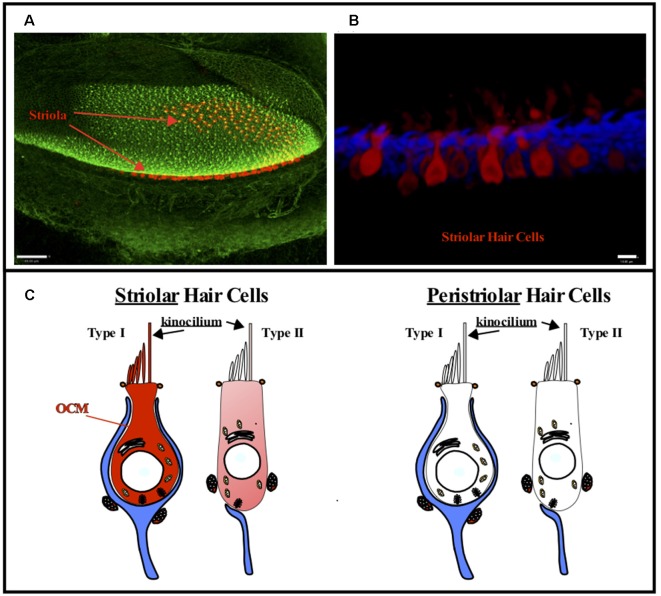
OCM expression in vestibular hair cells. OCM is differentially expressed in striolar vs. peristriolar hair cells. **(A)** A confocal image taken from a 1-month-old mouse utricle labeled for OCM (red) and phalloidin (green). Scale bar represents 50 μm. **(B)** A maximum intensity projection of OCM labeling (red) in an adult mouse utricular striola shows OCM labeling throughout the cytoplasm. The cells are predominately flask shaped representing Type I hair cells. Scale bar represents 10 μm. Modified from Simmons et al. (2010), permission granted by John Wiley & Sons, Inc. Phalloidin staining is shown in blue. **(C)** Schematic representation of Type I and Type II vestibular hair cells. OCM is highly expressed in Type I striolar hair cells and also expressed in some Type II striolar hair cells. In contrast, OCM is not expressed in peristriolar hair cells.

Thalmann and colleagues (Sakaguchi et al., 1998) first suggested OCM may be expressed in vestibular hair cells and this suggestion was later validated in all vestibular organs in both the mouse and rat by Simmons et al. (2010) and Hoffman et al. (2018). Further, Simmons et al. (2010) suggested that OCM expression was found in type-I vestibular hair cells in either striolar regions of macular organs (utricle and saccule) or central zone regions of the cristae (Figure 4). Similar to the cochlea, vestibular organs contain two types of hair cells, type I and type II, that have distinct shapes, innervation patterns, and differential distributions within each vestibular organ. Unlike adult cochlear hair cells, vestibular hair cells also have a true cilium called the kinocilium, which dictates the polarity or directional sensitivity of the hair cells. Also, in the utricle and saccule, the striola is a distinct region bordered by a line of polarity reversal (LPR) of the hair bundles, has an absence of CB-D29k and PVALB expression, and mostly composed of type I hair cells. Although OCM is expressed in type-I hair cells of the striola, PVALB is expressed in type-I hair cells of the surrounding, peristriolar region (Demêmes et al., 1993; Simmons et al., 2010). As shown in Figure 4, OCM expression within vestibular hair cells is not like the pattern observed in within cochlear OHCs. Vestibular hair cells show diffuse OCM labeling throughout the hair cell body and extending into the hair bundle including the kinocilium (Simmons et al., 2010; Hoffman et al., 2018). In the striolar region of the utricle, OCM is expressed in the majority of striolar type-I hair cells and is found in some type-II cells. Hoffman et al. (2018) recently determined that OCM is present in 83% of striolar type-I hair cells and only 23% of striolar type-II cells within the utricle (Figure 4). Unlike the cochlea, both OCM protein and mRNA expression in vestibular hair cells diminishes with age. Since there are known vestibular deficits that occur with aging, whether the absence of OCM enhances these vestibular deficits should be explored.

### Developmental Regulation of OCM in the Cochlea

The onset of OCM expression in the mouse cochlea occurs around P3–P4 and in the rat cochlea around P1–P2, but these postnatal days represent equivalent gestational lengths (Yang et al., 2004; Simmons et al., 2010). Yang et al. (2004) reported that OCM expression in OHCs coincides with the onset of efferent nerve innervation beginning around P2 and peaks around P10, before the onset of hearing. At no age did they find OCM mRNA or protein in IHCs, but readily found PVALB immunoreactivity in both IHCs and OHCs throughout the pre-hearing period. Further, OCM immunoreactivity was not detected in immature OHCs in culture although PVALB was readily observed under all culture conditions suggesting that PVALB and OCM are regulated differently. Hackney et al. (2005) measured the levels of OCM concentrations using immunogold techniques and found that OCM expression changed most dramatically from P7 to P26, far outstripping any residual expression of other CaBPs (Table 2). The cytoplasmic concentration of OCM varied in hair cells both as a function of frequency region and development. As given in Table 2, OCM expression decreased in low frequency, apical IHCs from ~400 μM at P7 to negligible levels in the adult, while it increased in OHCs from negligible levels at P7 to roughly 1.9 mM in low frequency OHCs and 2.9 mM in high frequency, basal OHCs. The difference between the two studies could simply be explained by technical differences between situ hybridization and immunogold labeling. Simmons et al. (2010) also investigated OCM mRNA and protein expression in P0–P10 mice. They showed that in basal regions of the P2–P4 cochlea, OCM protein was expressed only in OHCs, unlike the other three CaBPs, which showed immunostaining in both IHCs and OHCs. In one out of three mice, OCM was first visible in the base at P2 and present in basal regions of all mice tested by P4 with apical expression by P6. Additionally, OCM expression within the OHC shifted from a diffuse cytoplasmic staining pattern at P3 and P6, to a more polarized basolateral membrane pattern by P8 (Simmons et al., 2010).

Differing markedly from the pattern of OCM expression, other CaBPs such as PVALB, CB-D28k and CB-D29k are expressed prior to birth in rats and mice which is much earlier than OCM (Table 2). In mice, OCM, PVALB, CB-D28k, and CB-D29k mRNA and protein expression mirror those observed in the rat. In basal regions of the P8 cochlea, Simmons et al. (2010) showed robust OCM immunoreactivity in OHCs and diminishing immunoreactivity to PVALB, CB-D28k, and CB-D29k. Buckiová and Syka (2009) also investigated CB-D28k immunoreactivity in both the embryonic and postnatal murine cochlea with similar findings. Analyses of the developmental expression of OCM in rats and mice demonstrate an interesting pattern of staining along the cochlear length. OCM and other CaBPs are not expressed evenly from base to apex nor are they evenly expressed within the three rows of OHCs (Yang et al., 2004; Hackney et al., 2005; Simmons et al., 2010).

### Developmental Regulation of OCM in Vestibular Organs

As shown in Table 3, OCM immunoreactivity is present in mouse vestibular hair cells at embryonic day 18 (E18) and is absent after 10 months of age (Simmons et al., 2010). Indeed, utricles from animals from ages 6–8 months contain sparse OCM immunoreactivity, consistent with the progressive loss of OCM mRNA as the animals age. Although considerable studies have been done on the developmental expression of CaBPs in rodent vestibular organs (Dechesne and Thomasset, 1988; Dechesne et al., 1994; Zheng and Gao, 1997; Buckiová and Syka, 2009; Simmons et al., 2010), the relationship between OCM expression and the expression of other CaBPs in vestibular organs remains somewhat enigmatic. Differences in species, gestational ages, reagents, and experimental design vary between studies, which makes it difficult to pinpoint specific spatio-temporal expression patterns for the major CaBPs discussed in this review. For example, CB-28k and CB-29k appear in the vestibular hair cells as early as E17 but disappear in early postnatal development in some studies (Dechesne and Thomasset, 1988; Zheng and Gao, 1997; Buckiová and Syka, 2009), and around P12 in the case of CB-29k (Dechesne et al., 1994). CB-28k and CB-29k are not expressed in the vestibular hair cells of adult rodents but are expressed in calyceal afferent endings innervating type I hair cells (Desmadryl and Dechesne, 1992; Desai et al., 2005). PVALB expression is visible after CB-28k and CB-29k at E19 and maintained through P7 in rat vestibular hair cells (Zheng and Gao, 1997). In adult rodents, PVALB demonstrates strong immunoreactivity in the cell body and cuticular plates of type I hair cells of the peristriolar regions of the utricle (Demêmes et al., 1993). Within the striola, PVALB expression is restricted to the calyx type endings attached to the hair cells but absent from hair cell bodies (Simmons et al., 2010; Stone et al., 2018). Furthermore, OCM expression in hair cells of the striola may be independent of additional organizing phenotypes such as planar cell polarity (Simmons et al., 2010; Jiang et al., 2017; Hoffman et al., 2018). Missing from the literature are experiments that show co-localization of these proteins throughout development. It would be beneficial if OCM, PVALB, CB-D28k and CB-D29k proteins were analyzed in the vestibular organs of one rodent species from E17-P20. Careful consideration should be taken in distinguishing expression in cell body, hair cell bundles and calyceal ending, as well as striolar vs. peristriolar expression. The end result would be a powerful database for demonstrating the transitional expression patterns of the CaBPs as they are enlisted for essential processes across the different regions of these complex organs.

### OCM in Cochlear Function

As the dominant CaBP in OHCs, OCM has the potential to affect multiple pathways involving hair cell function and survival. A recent study of a near ubiquitous deletion of OCM demonstrated that OCM is not essential to cochlear development (Tong et al., 2016). However, using auditory brainstem response (ABR) and distortion product otoacoustic emission (DPOAE) measurements, Tong et al. (2016) show that *Ocm* knockout (KO) mice on the C57BL/6 genetic background exhibit progressive hearing loss starting at 1–2 months and are essentially deaf by 3–4 months. In the *Ocm* KO, progressive hearing loss at 2 months occurs prior to the loss of hair cells, suggesting that OCM may be protective against hearing loss (Tong et al., 2016). By 4 months, *Ocm* KO mice showed significantly greater OHC loss compared to age-matched WT mice. The eventual OHC loss suggests that OCM buffering capacity could also regulate cell survival. Additionally, *Ocm* KO mice showed altered expression characteristics for Prestin (Tong et al., 2016), a motor protein essential to OHC motility (Belyantseva et al., 2000; Zheng et al., 2000). Since DPOAEs are a byproduct OHC amplification (Gold, 1948; Kemp, 1978; Dallos, 1985) and Prestin is the motor protein required for OHC amplification, it is plausible that in the *Ocm* KO, OHCs have reduced DPOAEs due to a loss of OCM-regulated Prestin function. Overall, Ca^2+^ buffers do not appear to compensate for one another in the inner ear since PVALB and CB-D29k were not upregulated in OHCs in response to the loss of OCM (Tong et al., 2016) similar to previous reports in *Pvalb* KO mice (Chen et al., 2006). Similarly, a triple knock out of three Ca^2+^ buffers (*Pvalb*, *Calb1*, and *Calb2*) showed only minor impacts on hearing (Pangrsic et al., 2015), suggesting that these Ca^2+^ buffers may be unable to replace OCM function. Interestingly, OCM did appear to be upregulated in neuronal cells of Neurexin 1alpha/beta origin of *Pvalb* KO mice (Csillik et al., 2010). This upregulation suggests that in tissues outside of the inner ear, OCM has the potential to be expressed under certain conditions. However, because there have not been any follow-up studies, the extent of upregulation and the impact on neuronal function is still unknown. Thus, OCM is the primary CaBP in OHCs and seemingly functions in a unique manner relative to the other EF-hand CaBPs.

### OCM in Vestibular Function

As shown in Table 3, OCM is expressed earlier and downregulated sooner in the vestibular organs than in the cochlea. How might the restricted temporal and spatial expression of OCM in the striola affect overall vestibular function? Vestibular hair cells are turned over and replaced by the supporting cells in birds (Jørgensen and Mathiesen, 1988; Roberson et al., 1992; Tsue et al., 1994a,b; Kil et al., 1997) and mammals (Lin et al., 2011; Bucks et al., 2017). In mammals, while both type I and type II vestibular hair cells of the utricle are removed, only type II hair cells are replaced by the supporting cells under either damaging or non-damaging conditions (Bucks et al., 2017). Since OCM is predominantly expressed in type I vestibular hair cells of the striola (Hoffman et al., 2018), this leads to questions about how OCM expression may be involved in the functional distinctiveness of type I hair cells. Furthermore, is the loss of OCM expression in vestibular organs of older animals due to the loss of these specific hair cell types or do the cells die as a result of decreased OCM expression? Kirkegaard and Nyengaard (2005) did not observe any decrease in type I or type II hair cell numbers in adults up to 73 weeks old. However, they did not quantify differences in striolar vs. extrastriolar hair cell numbers, which would be particularly interesting given the restricted expression pattern of OCM to the striolar region. Further experiments are needed to determine the functional consequences of OCM expression in these hair cells. Some of these questions can be answered with tools already available. For example, striolar organization can be analyzed in young and old WT and *Ocm* KO mice to determine if OCM regulates the unique distribution of hair cell types in the vestibular organs. Additionally, vestibular function with direct behavioral measurements before and after hair cell loss and relative to OCM expression should be assessed. The *Ocm* KO mouse could be a corroborative model for these functional assessments.

## Oncomodulin in Immune Cells

OCM has been found in macrophages and neutrophils where there has been tissue damage. These leukocytes are involved in many critical biological processes, including inflammation and tissue regeneration and remodeling. Following tissue injury, dying cells trigger an inflammatory response that recruits neutrophils and macrophages (Wynn and Vannella, 2016; Vannella and Wynn, 2017). The most abundant white blood cell type, neutrophils, serve as first responders to inflammation. Attracted to sites of inflammation by chemotaxis, they cross through blood vasculature where they phagocytize foreign cells and degranulate destructive antimicrobial proteins. Bone marrow-derived monocytes arrive later to the site of inflammation where they are activated by cytokines and transform into a variety of macrophage phenotypes, including inflammatory (M1) and tissue repair (M2). M1 macrophages dominate the early inflammation response and either become M2 cells to promote regeneration and repair or are recruited as M2 macrophages. The vast nuances of activated-macrophage phenotypes are beyond the scope of this article and are reviewed in other excellent articles (Gordon and Taylor, 2005; Martinez and Gordon, 2014; Murray et al., 2014; Wynn and Vannella, 2016; Vannella and Wynn, 2017).

Benowitz and colleagues (Yin et al., 2006, 2009; Benowitz and Yin, 2008, 2010; Cui et al., 2009; Kurimoto et al., 2010, 2013; Benowitz and Popovich, 2011; de Lima et al., 2012; Siawaya et al., 2013; Marin et al., 2016) have extensively investigated the role of OCM in an inflammatory-mediated nerve regenerative model. The first report linking OCM to macrophage activity was using spontaneously immortalized NR8383 cells (Yin et al., 2006), a macrophage-like cell line isolated from normal rat alveolar lung tissue (Helmke et al., 1987). NR8383 cells express and secrete OCM, and the addition of the inflammatory agent zymosan significantly upregulates both expression and secretion of OCM (Yin et al., 2006). Additionally, cell culture media from zymosan-stimulated NR8383 cells (MCM) confers pro-regenerative effects to mouse retinal ganglion *ex vivo* cultures (Yin et al., 2006). However, OCM does not inherently confer enhanced regenerative properties across all cell types. In an experiment using 3-day pancreas explants from E13 rats, treatment with OCM or MCM suppresses gene expression of pancreas duodenum homeobox-1 (Pdx-1) and neurogenin3 (Ngn3), two genes with significant roles in the development of the pancreas (Siawaya et al., 2013). Further *in vivo* studies in the pancreas are warranted, but at the very least, OCM cannot be assumed to possess universal pro-regenerative capacities.

*In vivo* expression of OCM was demonstrated in infiltrating macrophages of the aqueous and vitreous humor of mice following intra-ocular inflammation. This expression was elucidated *via* co-labeling immunofluorescence experiments using *ex vivo* retinal ganglion cultures with antibodies against CD68 and OCM (Yin et al., 2009; Kurimoto et al., 2010). Furthermore, the onset and variation of OCM expression in the eye is well documented. Expression of OCM mRNA in the infiltrative cells, which include CD68+ macrophages, that enter the mouse vitreous humor peaks at around day 1 following lens injury or zymosan injection and begins to decline by day 3 (Yin et al., 2009). In retinal tissue, expression of OCM mRNA remains low, though levels of the protein increase markedly thus implicating invading immune cells for the expression and secretion of OCM as opposed to resident cells (Yin et al., 2009; Kurimoto et al., 2013). OCM binds to the inner retina in mice and to isolated retinal ganglion cells in rats in a cAMP-dependent manner using either zymosan stimulation or the addition of exogenous cAMP (Yin et al., 2006, 2009; Kurimoto et al., 2010). However, there are contrasting reports suggesting OCM is not a significant factor in nerve regeneration. One such report did not find OCM expressed in macrophages post-lens injury (Hauk et al., 2008), and there are two studies that suggest depleting macrophages, and consequently OCM does not significantly inhibit nerve regeneration (Müller et al., 2007; Hauk et al., 2008). Although these studies dispute OCM participation in inflammation-induced regeneration, more recent experiments seem to confirm its role in the process. For example, studies of zymosan-induced secretion of OCM *in vivo* repeatedly demonstrate that OCM facilitates neuron regeneration. How much of an impact OCM has on macrophage-induced nerve repair can only be resolved with studies involving animal models with an OCM targeted deletion.

## Oncomodulin in Cancer and Development

Initially, the discovery of OCM in several types of mouse, rat, and human tumors made OCM attractive as a potential cancer marker (Boynton et al., 1982; MacManus et al., 1982). Throughout the 1980s and early 1990s there were several studies aimed at evaluating OCM expression in various tumor types. Diethylnitrosamine is a potent carcinogen that causes liver cancer and was used to induce hepatocarcinomas that express OCM compared with normal liver tissue (Bernaert et al., 1989). OCM is expressed in human tumor biopsies (Levine et al., 1987) and solid tumors were generated in nude rats by injection of transformed cell lines, cell lines derived from human cancers, and extracts from human and rat tumors (MacManus et al., 1982, 1984; MacManus, 1984). Significantly, the expression of OCM was not detected in normal tissues and was almost always expressed in matched tumor types. However in some cases, non-specific antibody staining sometimes made it appear that normal tissues were positive for anti-OCM reactive species (Levine et al., 1987), and not all human tumor cell lines test strongly for OCM (Huber et al., 1990) as in rodent counterparts. In the rat, OCM is under the control of a strong upstream LTR promoter (Banville and Boie, 1989; Furter et al., 1989; Rentsch et al., 2006) but not in the mouse (Banville et al., 1992), which could contribute to significant differences in expression of the protein in tumors and cell lines derived from their tissues. Conflicting OCM expression in rodent and human primary tumor analyses may be why interest in this protein as a potential cancer marker waned. Thus, the term “oncomodulin” may be a misnomer and a more suitable name should be considered.

While OCM does not induce transformation of cultured cells (Mes-Masson et al., 1989), several studies documented a clear increase in the expression of OCM in neoplastic transformation of rodent cell lines (MacManus et al., 1982, 1983a,b; Brewer et al., 1984; Pfyffer et al., 1984; Brewer and MacManus, 1987; Sommer and Heizmann, 1989; Sommer et al., 1989). Unlike calmodulin which may be upregulated nearly ubiquitously in transformed cells, the *de novo* synthesis of OCM appears more selective (MacManus et al., 1982; Sommer and Heizmann, 1989; Sommer et al., 1989). For example, chemical transformation of rat fibroblast cell lines almost always leads to enhanced expression of calmodulin, but *de novo* expression of OCM is seen only in a subset of transformed cell lines whereas it is not seen in non-transformed cell lines (Sommer et al., 1989; Klug et al., 1994). These facts suggest OCM expression may be suppressed in non-transformed cells but can be upregulated in transformed cells. As with the case of *Pvalb* KO mice, extenuating circumstances seem to be required to actively synthesize OCM outside of the inner ear after postnatal development, which makes OCM distinct from other CaBPs.

In order to determine its function in tumors, early studies purified OCM from various tumors and investigated the effect of OCM on glutathione reductase (Palmer et al., 1990), cyclic nucleotide phosphodiesterase (MacManus, 1981; Mutus et al., 1985), and cell-cycle regulation (Boynton et al., 1982). Early *in vitro* studies of OCM suggest that it could be involved in the regulation of glutathione reductase, an enzyme antioxidant responsible for maintaining reducing conditions within cells (Palmer et al., 1990). This study showed that in the presence of Ca^2+^, purified OCM inhibited glutathione reductase. They speculated that increasing intracellular levels of glutathione could enhance the overall reducing environment, thus protecting cells from oxidative stress. There have been no follow-up studies to confirm whether OCM inhibition of glutathione reductase occurs intracellularly or whether OCM protects against oxidative stress. However, this early study is consistent with a more recent study by Permyakov et al. (2014, 2017) that suggests the parvalbumin family of proteins have strong antioxidant properties.

There was also early interest in whether OCM affected levels of cyclic nucleotide phosphodiesterase (MacManus, 1981; Mutus et al., 1985; Palmer et al., 1990). Phosphodiesterases are enzymes that degrade cAMP and cGMP, intracellular signaling molecules key to numerous cellular functions. Although a common observation with calmodulin, parvalbumins are not known to stimulate phosphodiesterases and this feature would further distinguish OCM. However, these early reports of OCM modulating phosphodiesterases remain controversial and unsubstantiated. Subsequent studies by Klee and Heppel (1984) and Clayshulte et al. (1990) failed to show any modulatory effect of OCM on cyclic nucleotide phosphodiesterases. Thus, evidence is lacking that OCM has any significant effect on phosphodiesterases and whether it is physiologically relevant.

Several studies showed that raising the Ca^2+^ concentration, or adding OCM, stimulated DNA synthesis in non-neoplastic rat liver cells whose DNA-synthetic ability had been reduced by incubation in low Ca^2+^ media (Boynton et al., 1982; MacManus et al., 1982). These studies suggested that there was a Ca^2+^ OCM complex since the action of OCM was blocked when Ca^2+^ concentrations were either reduced or blocked by trifluoperazine. Since OCM was not found in regenerating liver, it made the interpretation of these results difficult.

Expression of OCM in tumors also led to questions about the non-cancer function of the protein. Prior to its discovery in inner ear tissues, the only normal tissues with OCM expression were in the pre-implantation embryo and placental tissue during fetal development. MacManus et al. (1990) are credited with documenting OCM immunoreactivity and mRNA in the morula and blastula stages of rat embryos. The extra-embryonic expression of OCM placental tissues was found in both rat (Brewer and MacManus, 1985; MacManus et al., 1985; Gillen et al., 1988; Brewer et al., 1989) and human (MacManus et al., 1985; Brewer and MacManus, 1987; Föhr et al., 1993). However, OCM expression in placental tissues is restricted to the invasive cytotrophoblasts. No further analyses on the function of OCM during embryonic development exist. The finding of OCM expression in tumors and placental cells raises questions about what these cell types have in common. Obviously, placental cells and tumors are mitotically active. Does OCM play a different role in tumor and cytotrophoblasts cells compared to roles of OCM in either hair cells or immune cells?

## Proposed Models of Oncomodulin Function

Based on five decades of research, OCM has been implicated in at least three functional roles: the first proposed role was as an agent that induces DNA synthesis in cells associated with proliferation in tumors or during development; second, as a distinct mobile Ca^2+^ buffer found only in subsets of hair cells and associated with hearing protection; and third, as a secreted factor by macrophages and neutrophils that promotes nerve regeneration. Such widely distinct functional roles seem unusual for such a small protein with extremely limited tissue distribution. For example, PVALB is expressed in diverse tissues, ranging from skeletal muscle to inhibitory neurons and yet, is believed to have a single mechanism by which it functions. By comparison, the role of OCM remains enigmatic. Based on work over the last two decades, we propose two widely differing models of OCM function below.

### Model for OCM Function in Hearing

**Figure 5:**
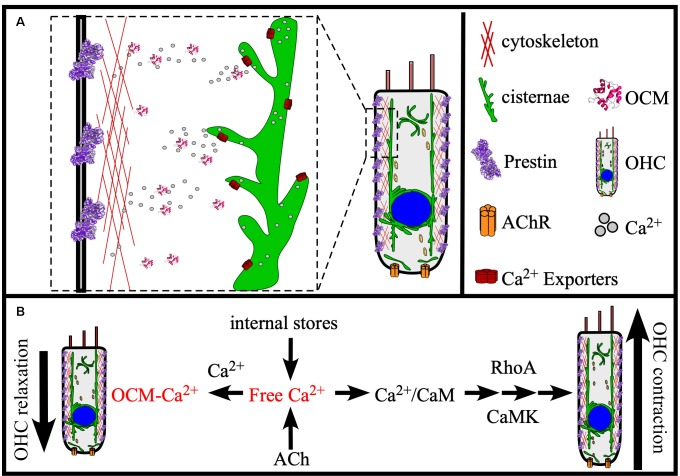
Model of OCM function in the cochlea. **(A)** OCM is preferentially localized to the basolateral membrane of OHCs where proteins involved in electromotility are also localized, like Prestin and Actin. At this location, OCM is readily available to modulate Ca^2+^ required for electromotility. Major sources of Ca^2+^ for OHCs are internal stores, such as the endoplasmic reticulum-like membranes termed cisternae that localize in patches along all borders of the cell, the Golgi-like compartment called Hensen bodies, and influx into the cell from the extracellular environment by ACh receptors (AChR). **(B)** Ocm can potentially regulate OHC function in OHC contraction and relaxation through at least two Ca^2+^-dependent mechanisms: (1) Modulating Ca^2+^ transients required for RhoA-dependent Actin polymerization; and (2) Modulating Ca^2+^ transients required for Ca^2+^/CaM-dependent Prestin stiffness/rigor. These Ca^2+^ pathways are essential to OHC elongation (contraction) and shortening (relaxation), and we propose that OCM helps regulate these mechanisms necessary for OHC stiffness and electromotility.

In the adult mammalian ear, OCM serves as the dominate EF-hand CaBP in OHCs and its absence leads to OHC dysfunction as inferred from diminished DPOAEs (Hackney et al., 2005; Simmons et al., 2010; Tong et al., 2016). Therefore, OCM may be critical for helping OHCs maintain Ca^2+^ signaling pathways key to OHC function. If OCM is present at the basolateral membrane of OHCs as suggested by previous reports (Simmons et al., 2010), then it may associate with proteins of the actin cortical lattice involved in OHC amplification and motility mechanisms as suggested by Tong et al. (2016). Following that suggestion, we propose that OCM helps to regulate OHC elongation (contraction) and shortening (relaxation) mechanisms associated with the cortical lattice. It is the cortical lattice that determines OHC stiffness and electromotility (Frolenkov et al., 2000). As illustrated in Figure 5, OCM can potentially modulate OHC stiffness and elongation through at least two Ca^2+^-dependent mechanisms: (1) regulation of Ca^2+^ transients that modulate Rho-dependent actin polymerization and hair cell elongation (Narumiya and Thumkeo, 2018); and (2) regulation of Ca^2+^ transients critical for Ca^2+^/CaM-dependent interactions with Prestin and/or hair cell stiffness (Keller et al., 2014). In any case, Ca^2+^ entry through nicotinic acetylcholine (ACh) receptors or L-type Ca^2+^ channels lead to Ca^2+^ transients amplified by Ca^2+^-induced Ca^2+^ release (CICR) from nearby cisternae, which increases intracellular levels of Ca^2+^. In addition to the amount of CICR, the magnitude and duration of these Ca^2+^ transients would be a function of cytosolic buffers and uptake into organelles (e.g., mitochondria and cisternae) through Ca^2+^ transporters. Importantly, the effects of OCM buffering would depend on its binding kinetics. If OCM is a very fast buffer, then it would decrease the amplitude of the Ca^2+^ transient and slow its decay. However, as a slow buffer, OCM would bind Ca^2+^ more slowly, having less of an impact on the amplitude of the Ca^2+^ transient, but a larger effect on its decay. Such is the case for PVALB in skeletal muscle, which binds Ca^2+^ slowly and therefore has minimum impact on the amplitude of Ca^2+^ transients, but significantly increases the speed of its decay contributing to the increased rate of relaxation (Racay et al., 2006). A recent report by Ranum et al. (2019) bolsters the view that OCM plays a major role in Ca^2+^-based mechanisms of elongation and stiffness. Ranum et al. (2019) identified *Ocm*, *Sri* (Sorcin) and *Slc26a5* as the top three genes that define the OHC transcriptome. Similar to OCM, Sorcin is an EF-hand CaBP localized throughout the body and lateral membrane of OHCs and is speculated to function in CICR from the OHC cisternae. If OCM has a similar role in OHCs as PVALB does in skeletal muscle, then it makes sense that OCM would work synergistically with Sorcin and other CICR Ca^2+^ regulators, or through regulating the Ca^2+^ signals induced by ACh.

### Model for OCM Function in Nerve Regeneration

**Figure 6:**
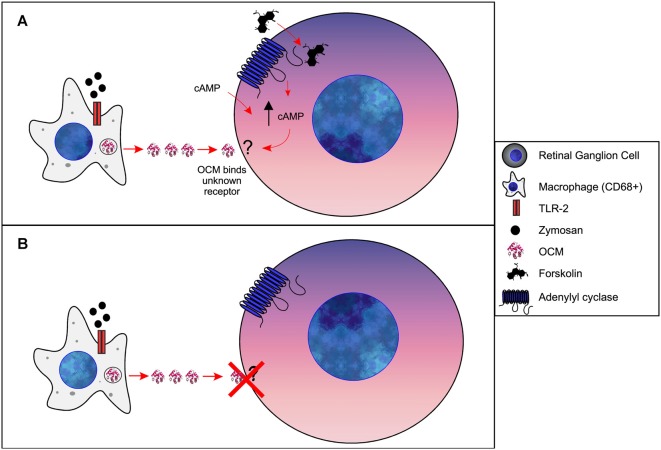
Model of OCM in immune cells of retina. **(A)** Forskolin causes an increase [cAMP]_i_ in retinal ganglion cells (RGCs). *Via* an unknown mechanism, increased [cAMP]_i_ results in OCM binding to an unidentified receptor. Administration of exogenous cAMP also results in OCM binding to RGCs. **(B)** Without increased [cAMP]_i_ OCM does not bind to RGCs.

A general hypothesis of OCM-induced regeneration was laid out by a series of experiments from Kurimoto et al. (2010). Briefly, these studies show that zymosan leads to increased levels of OCM immunoreactivity in the retina, which can be suppressed by the addition of a peptide fragment corresponding to OCM but not by an PVALB peptide fragment. Building on these experiments, they demonstrated that zymosan injection leads to increased retinal ganglion cell neural regeneration and is enhanced by the addition of a cAMP analog. Furthermore, gains in nerve regeneration were severely undercut when the OCM peptide fragment was added. Importantly, an OCM receptor and an exact signaling pathway have not been defined. Therefore, based on such studies, we propose the following model of OCM signaling in macrophages and nerve regeneration, as shown in Figure 6. When expressed in macrophages, OCM is upregulated and secreted as part of a response to inflammatory events whereby it augments an axonal regenerative response. Initial studies utilized the bacterial glycan zymosan, but neuronal lesions have been shown to produce this response as well. Once secreted, OCM either binds to a hypothetical surface receptor or enters neurons *via* an endocytic mechanism, but only when intracellular cAMP is elevated above basal levels. The mechanism detailing how elevated intracellular cAMP causes OCM binding is unknown.

## Conclusions

In this review, we show that OCM is evolutionarily distinguished from the majority of lower vertebrate β-parvalbumins. Experiments to date suggest that OCM may have an ambiguous function that depends upon the cell type in which it is expressed, and this ambiguity further distinguishes it from other EF-hand CaBPs. In sensory cells, recent studies suggest that OCM plays an essential role in maintaining auditory function, most likely affecting OHC motility mechanisms. In immune cells, OCM may be secreted in response to inflammatory signals and facilitates axon regeneration. Although originally defined as a potential oncoprotein, whether it plays a major role in this regard remains inconclusive. As such, the term “oncomodulin” may be a misnomer for mammalian β-parvalbumin. Independent of any oncogenic function, the process of transforming certain cells can lead to the *de novo* synthesis of OCM just as loss of PVALB can lead to upregulation of OCM in certain brain regions. All of these proposed roles for OCM beg the question whether it is possible for a single small molecule to function in such widely disparate ways. Our proposed models of OCM function are based on data from cochlear hair cells and retinal macrophages that present very divergent mechanisms of OCM function. Fully delineating the molecular details of how OCM protects adult auditory function and augments nerve regeneration will be essential next steps.

## Author Contributions

LC, AC and DS wrote the manuscript. AC and TR performed the phylogenetic analysis. All authors contributed to commenting and editing of the manuscript.

## Conflict of Interest

The authors declare that the research was conducted in the absence of any commercial or financial relationships that could be construed as a potential conflict of interest.
