# Supplementary material for: Oncomodulin: The Enigmatic Parvalbumin Protein
Source: Front Mol Neurosci. 2019 Oct 9;12:235. doi: 10.3389/fnmol.2019.00235 (PMC6794386; doi:10.3389/fnmol.2019.00235)
Supplement: Supplementary file 1 [file Presentation_1.pdf]

APPENDIX

H. sapiens **oncomodulin and α-parvalbumin homologs from protein BLAST (BLASTp) search**

BLASTp search sequences
>sp|P0CE72|ONCO_HUMAN Oncomodulin-1 OS=Homo sapiens GN=OCM PE=1 SV=1
MSITDVLSADDIAAALQECRDPDTFEPQKFFQTSGLSKMSANQVKDVFRFIDNDQSGYLDEEELKFFLQKFES
GARELTESETKSLMAAADNDGDGKIGAEEFQEMVHS

>sp|P20472|PRVA_HUMAN Parvalbumin alpha OS=Homo sapiens GN=PVALB PE=1 SV=2
MSMTDLLNAEDIKKAVGAFSATDSFDHKKFFQMVGLKKKSADDVKKVFHMLDKDKSGFIEEDELGFILKGFS
PDARDLSAKETKMLMAAGDKDGDGKIGVDEFSTLVAES

BLASTp search parameters
To reduce the probability of junk sequences being returned, the BLASTp search parameters were set to default except for the following:

- -Search RefSeq proteins only
- -Excluded models (XM/XP) and non-redundant RefSeq proteins (WP)
- -Filtered low complexity regions
- -Do not make compositional adjustments to the scoring parameters.

Dealing with duplicate sequences in BLASTp results
From the returned results, there were some oddities. Some sequences were returned in replicates that were ≥98% identical, i.e. *H. sapiens* oncomodulin and oncomodulin-2 sequences which differ at two amino acid sites. In these instances, the duplicate sequence that was least similar to the query was excluded. Contrarily, some sequences were annotated with the same name and species, but the amino acid compositions were highly dissimilar, i.e. two *Xenopus tropicalis* oncomodulin sequences deposited by the same research team in 2016. In these cases, all the duplicates were retained for the analysis.

Establishing a cut-off for selected sequences
Sequence selection criteria were based on E-values and species relevance. There was a significant drop-off in E-values corresponding to proteins not belonging to the parvalbumin family with one exception, a 134aa-long protein aptly named parvalbumin-like EF-hand containing protein (PVALEF) from *H. sapiens*. An alignment between this protein and *H. sapiens* OCM produced an E-value significantly higher than the other parvalbumins selected for the analysis, 7.44E-04 versus 3.61E-16, but also significantly lower than the non-parvalbumin sequence from non-vertebrates that were listed as the next best alignment (caltractin from *Zea mays*, E-value = 0.012), thus making PVALEF a unique intermediate hit. PVALEF was selected based on its similarity to parvalbumins in sequence length, composition (EF-hand containing) and species relevance. Aside from this instance, all other hits used in the analysis had an E-value threshold of ≤E-16.

Reducing redundant sequences from the two searches

As would be expected, the two independent BLASTs produced a high-degree of overlap of hits. These duplicates were eliminated prior to analyzing the sequences in MEGA7. Afterward, a final total of 50 sequences remained for the analysis, representing Classes *Actinopterygii, Chondrichthyes, Amphibia, Aves,* and *Mammalia*.

Including a root

In order to draw evolutionary conclusions, an out-group sequence was included in the analysis. In general, the out-group sequence should be a distant but similar protein to the sequences used in the analysis. Therefore, the protein sequence calmodulin, an EF-hand superfamily protein, was used from the non-vertebrate organism *Plasmodium falciparum*. The rooting sequence was included with the 50 BLAST sequences in the alignment and model analysis.

Aligning the sequences

The sequences were imported in MEGA7—a powerful sequence analysis software containing alignment algorithms, sequence evolution model testing, and phylogenetic tree generating algorithms. They were aligned using the algorithm, MUSCLE, using the default settings.

Testing the correct evolutionary model to use for generating the tree

MEGA7 can determine which model best fits the aligned sequences for constructing the maximum likelihood (ML) phylogenetic tree. This best model test was performed using the following settings: Tree to Use = Neighbor-joining tree, Statistical Method = ML, Gaps/Missing Data Treatment = Partial deletion with a site coverage cutoff = 95%, and Branch Swap Filter = none. In total, 56 models were tested. Models with the lowest Bayesian Information Criterion (BIC) score are considered the best fit. The model with the lowest score from the test was the LG+G+I model with a BIC score of 9469.225.

Constructing the ML tree

We asked MEGA7 to construct a ML phylogenetic tree using the LG+G+I model and to bootstrap the tree with the highest log likelihood for 500 replications. All other parameters were left at default.
